# Anti-inflammatory and antioxidant effects of *Pogostemon stellatus* (Lour.) Kuntze via MAPK, NF-κB, and Nrf2 signaling pathways in LPS-activated RAW 264.7 macrophages

**DOI:** 10.3389/fphar.2025.1679919

**Published:** 2025-09-25

**Authors:** Haelim Yoon, You Li Gong, Jihye Seo, Jiyu Kim, Md. Salah Uddin, Sang Beom Han, Sayeon Cho

**Affiliations:** ^1^ Laboratory of Molecular and Pharmacological Cell Biology, College of Pharmacy, Chung-Ang University, Seoul, Republic of Korea; ^2^ Laboratory of Biomedical Mass Spectrometry, College of Pharmacy, Chung-Ang University, Seoul, Republic of Korea; ^3^ Botanika, Botanical Research Centre, Dhaka, Bangladesh

**Keywords:** Pogostemon stellatus (Lour.) Kuntze, plant extract, nitric oxide, prostaglandin E2, cytokine, tumor necrosis factor-α, lipopolysaccharide

## Abstract

**Background:**

*Pogostemon stellatus* (Lour.) Kuntze is a plant native to South and East Asia belonging to the Lamiaceae family, whose members are widely recognized for their anti-inflammatory and antioxidant properties. However, these properties have not been extensively studied in *P. stellatus* (Lour.) Kuntze. This study aimed to evaluate the anti-inflammatory and antioxidant effects of methanol extract of *P. stellatus* (Lour.) Kuntze (MPSK) in LPS-stimulated RAW 264.7 macrophages.

**Methods:**

RAW 264.7 macrophages were stimulated with LPS and treated with MPSK. Anti-inflammatory effects were assessed by measuring nitric oxide (NO) and prostaglandin E_2_ (PGE_2_) levels and the expression of inducible NO synthase (iNOS), cyclooxygenase-2 (COX-2), interleukin-6 (IL-6), interleukin-1β (IL-1β), and tumor necrosis factor-α (TNF-α) at both mRNA and protein levels. Nuclear factor-κB (NF-κB) and mitogen-activated protein kinase (MAPK) pathway involvement was examined. Antioxidant capacity was determined using a DPPH radical scavenging assay, and nuclear factor erythroid 2-related factor 2 (Nrf2) and its downstream targets were evaluated.

**Results:**

MPSK markedly attenuated the production of inflammatory mediators and cytokines in LPS-induced RAW 264.7 macrophages. NO production was suppressed by 98%, compared with the LPS-treated group. PGE_2_ production was also inhibited by 64%. Consistent with these findings, MPSK significantly reduced the protein and mRNA expression levels of iNOS and COX-2. Furthermore, MPSK suppressed the production and expression of pro-inflammatory cytokines, including IL-1β, IL-6, and TNF-α. Notably, when MPSK was administered at 100 μg/mL, IL-6 and IL-1β production were reduced by 21% and 18%, respectively, compared with the LPS-treated group. These anti-inflammatory effects were associated with the inhibition of NF-κB and MAPK signaling pathways. Immunoblot analysis demonstrated that NF-κB protein expression was reduced by 72% upon treatment with MPSK, while the phosphorylation of ERK, JNK, and p38 was suppressed by 38%, 56%, and 35%, respectively. MPSK suppressed ROS levels in RAW 264.7 cells by 62%, demonstrating its potent antioxidant capacity. This antioxidant activity of MPSK was demonstrated by the upregulation of Nrf2 and its downstream antioxidant target genes.

**Conclusion:**

MPSK possesses both anti-inflammatory and antioxidant activities through regulation of NF-κB, MAPK, and Nrf2 pathways, suggesting its potential as a therapeutic agent against inflammation and oxidative stress.

## 1 Introduction

Inflammation is a complex defense mechanism that responds to external stimuli such as infections or tissue damage. However, chronic or prolonged inflammation is recognized as a key contributor to the development of various diseases, including cardiovascular disorders, diabetes, and cancer (C.-H. Lee and Choi, 2018). In recent years, research has increasingly focused on regulating inflammatory responses through molecular signaling pathways, with the goal of developing therapeutics that target specific inflammatory mediators. Inflammatory responses are mediated by various immune cells, including T cells and B cells (Chen et al., 2018). Among these, macrophages play a critical role as the first line of defense against infection and tissue injury. They remove pathogens and cellular debris through phagocytosis and secrete a range of inflammatory mediators that activate other immune cells (C.-H. Lee and Choi, 2018; Chen et al., 2018). As a key component of both the initiation and resolution phases of inflammation, activated macrophages amplify inflammatory responses by releasing pro-inflammatory cytokines (Chen et al., 2018; C.-H. Lee and Choi, 2018).

Lipopolysaccharide (LPS), a major component of the outer membrane of Gram-negative bacteria, is a potent activator of the immune system and is commonly used to induce inflammation in experimental models (Page et al., 2022). The RAW 264.7 macrophage cell line is frequently employed in *in vitro* studies of inflammation due to its strong response to LPS stimulation (M. Kim et al., 2024; Wei et al., 2022). In LPS-stimulated RAW 264.7 macrophages, the expression of inducible nitric oxide synthase (iNOS) and cyclooxygenase-2 (COX-2) is closely associated with the production of inflammatory mediators such as nitric oxide (NO) and prostaglandin E_2_ (PGE_2_) (D. H. Kim et al., 2013; Kim et al., 2007). LPS is primarily recognized by Toll-like receptor 4 (TLR4) on immune cells, including macrophages, initiating intracellular signaling cascades that trigger inflammatory responses (Soares et al., 2010). Upon activation, TLR4 recruits adaptor proteins such as Myeloid differentiation primary response 88 (MyD88), which subsequently activates TNF receptor-associated factor 6 (TRAF6) (Soares et al., 2010). TRAF6 then stimulates key signaling pathways, including nuclear factor-kappa B (NF-κB) and mitogen-activated protein kinase (MAPK) pathways.

The NF-κB signaling pathway is activated through TRAF6-mediated stimulation of transforming growth factor-β-activated kinase 1 (TAK1) and the inhibitor of NF-κB (IκB) kinase (IKK) complex (Soares et al., 2010). The IKK complex consists of IKKα, IKKβ, and the regulatory subunit IKKγ (also known as NEMO) (Liu et al., 2017). Among these, IKKβ plays a central role in phosphorylating IκBα, marking it for ubiquitination and subsequent degradation by the proteasome. Under resting conditions, NF-κB, typically a p65 (RelA)/p50 heterodimer, is sequestered in the cytoplasm by IκBα (Liu et al., 2017). Upon IκBα degradation, NF-κB translocates into the nucleus, where it binds to promoter regions of inflammatory genes to drive transcription (Liu et al., 2017).

Furthermore, TAK1 activates downstream MAPKs, including p38, c-Jun N-terminal kinase (JNK), and extracellular signal-regulated kinase (ERK) (Park et al., 2024). In turn, these activated MAPKs activate the transcription factor activator protein-1 (AP-1) in the cytoplasm, which subsequently binds to the promoter regions of inflammatory genes and enhances the expression of pro-inflammatory cytokines such as tumor necrosis factor-α (TNF-α), interleukin-1β (IL-1β), and interleukin-6 (IL-6) by binding to their gene promoters (Park et al., 2024; Zhang and An, 2007).

Upon exposure to inflammatory stimuli, macrophages produce reactive oxygen species (ROS) as part of the host defense response. However, excessive ROS accumulation can lead to oxidative stress, contributing to the pathogenesis of chronic inflammatory diseases (Canton et al., 2021; Yu et al., 2024). To counteract this, macrophages activate endogenous antioxidant defense mechanisms that regulate intracellular ROS levels (Canton et al., 2021). A central component of this antioxidant response is nuclear factor erythroid 2-related factor 2 (Nrf2), a transcription factor that maintains cellular redox homeostasis. Nrf2 binds to the antioxidant response element (ARE) in the promoter regions of target genes, inducing the expression of antioxidant enzymes such as heme oxygenase-1 (HO-1) and NAD(P)H quinone oxidoreductase-1 (NQO1) (Canton et al., 2021). Under normal physiological conditions, Nrf2 is retained in the cytoplasm by Kelch-like ECH-associated protein 1 (Keap1), which facilitates its ubiquitination and subsequent proteasomal degradation. In response to oxidative stress, Keap1 undergoes conformational or oxidative modifications that disrupt its ability to target Nrf2 for degradation. As a result, Nrf2 accumulates, translocates into the nucleus, and activates the transcription of antioxidant genes. Inadequate Nrf2 activity in macrophages leads to excessive ROS accumulation and increased production of inflammatory cytokines. Therefore, pharmacological activation of Nrf2 is a promising therapeutic approach for modulating macrophage function and suppressing inflammation (Canton et al., 2021).

*Pogostemon stellatus* (Lour.) Kuntze, a member of the Lamiaceae family, was formerly known as Dysophylla Stellata Benth. Ex Wall (2025). The plant is an annual herb widely distributed across parts of South and East Asia, including Bangladesh, Cambodia, Korea, China, and Japan, as well as Australia (Dysophylla Stellata Benth. Ex Wall, 2025). It has also been reported to grow wild in Taiwan and Jeju Island, Korea (Dysophylla Stellata Benth. Ex Wall, 2025). *Pogostemon stellatus* (Lour.) Kuntze has upright stems that grow submerged or above water, producing whorls of four to eight linear or lanceolate leaves (Gautam et al., 2010). This plant grows in various forms in the wild and cultivated environments, producing purplish-red flowers and small, oval-shaped nuts (Merlin and Franco, 2009). Traditionally, its flowers have been used by India for the reduction of fever (Merlin and Franco, 2009). However, ethnopharmacological reports on this species remain scarce, and direct scientific evidence supporting its medicinal properties is limited. However, other members of the Lamiaceae family are well known for their antioxidant and anti-inflammatory activities (Kianmehr et al., 2023; Jasemi et al., 2022). Among them, *Pogostemon cablin* (Blanco) Benth. (commonly known as patchouli) has been extensively studied for its anti-inflammatory activities (Wu et al., 2025; Jeong et al, 2013). *Pogostemon cablin* (Blanco) Benth contains diverse bioactive compounds, including terpenoids, phytosterols, flavonoids, and organic acids, and its anti-inflammatory effects have been demonstrated in various studies, including those utilizing RAW 264.7 macrophage cells (Wu et al., 2025). *Pogostemon stellatus* (Lour.) Kuntze, a member of the Lamiaceae family, is expected to possess notable biological activities, similar to other well-studied species within the same family. Preliminary studies have indicated that *P. stellatus* (Lour.) Kuntze may exert anti-inflammatory effects through its antioxidant constituents, such as flavonoids and phenolic compounds (Gautam et al., 2011; Silva et al., 2016). However, its anti-inflammatory activity has only been supported by limited *in vitro* evidence, primarily from assays measuring NO production. Moreover, the molecular mechanisms by which *P. stellatus* (Lour.) Kuntze exerts these effects, particularly in LPS-stimulated RAW 264.7 macrophages, remain largely unknown.

Therefore, the present study sought to evaluate the anti-inflammatory potential of the methanol extract of *P. stellatus* (Lour.) Kuntze (MPSK) and to investigate whether its effects are mediated through the NF-κB and MAPK signaling pathways, as well as the Nrf2-mediated antioxidant defense system. These findings may support the potential use of *P. stellatus* as a functional bioactive material and provide a scientific basis for its application in the development of therapeutic agents for inflammatory diseases.

## 2 Materials and methods

### 2.1 MPSK preparation and extract construction

The plant extract of *P. stellatus* (Lour.) Kuntze (FBM274-067) was obtained from the International Center for Biomaterials, Korea Research Institute of Bioscience and Biotechnology (KRIBB, Daejeon, Republic of Korea). *Pogostemon stellatus* (Lour.) Kuntze (KRIB 0069107) were collected from the Noakhali district, Chittagong province, Bangladesh, and stored in the KRIBB herbarium. *Pogostemon stellatus* (Lour.) Kuntze was extracted using methanol, a polar solvent used as an experimental standard solvent for highly efficient extraction of various physiologically active substances from plants. The specific extraction method is as follows. Shade-dried leaves and shoots (69 g) were ground and extracted with 1 L of 99.9% methyl alcohol (HPLC grade) using an ultrasonic extractor (SDN-900H, SD-ULTRASONIC CO., LTD.) at room temperature (RT). The sonication was performed for 15 min per cycle and repeated 30 times (total 120 min) at 40 kHz and 1500 W. The extract was filtered using a qualitative filter (No. 100, HYUNDAI MICRO CO., LTD.) and concentrated under reduced pressure, yielding 9.38 g of the dried extract (designated as MPSK). For the experiments, MPSK was dissolved in dimethyl sulfoxide (DMSO; Sigma-Aldrich, St. Louis, MO, United States), with the final DMSO concentration in culture media kept below 0.1%.

### 2.2 Cell culture

RAW 264.7 macrophages (ATCC, VA, United States) and human embryonic kidney 293 (HEK 293) cells were cultured in Dulbecco’s Modified Eagle’s Medium (DMEM; Invitrogen, CA, United States) supplemented with 10% fetal bovine serum (FBS; Invitrogen, CA, United States) and 1% penicillin-streptomycin (Life Technologies, NY, United States). Cells were maintained at 37 °C in a humidified incubator with 5% CO_2_. Cells were seeded and pretreated with MPSK (100 μg/mL) or vehicle control (DMSO) for 2 h. Following pretreatment, the cells were stimulated with either LPS (1 μg/mL) or PMA (50 nM).

### 2.3 LC-MS/MS system and data analysis

Quantitative analysis was performed using an Agilent 6495 triple quadrupole tandem mass spectrometry system coupled with an Agilent 1290 Infinity LC system (Agilent Technologies, Santa Clara, CA, United States). Data acquisition parameters and multiple reaction monitoring (MRM) transitions were optimized using the MassHunter Workstation software (version B.10.00; Agilent Technologies). Data processing was used to optimize the data acquisition parameters and MRM transitions. Data analysis was performed using the MassHunter Workstation Qualitative Analysis 10.0 and MassHunter Workstation Quantitative Analysis 10.1 software (Agilent Technologies). A Vortex-Genie 2 mixer (Scientific Industries, Bohemia, NY, United States) was used during sample preparation.

All standards were initially prepared as stock solutions in methanol at a concentration of 1 μg/mL. A mixed standard stock solution containing eugenin, luteolin, apigenin-7-glucoside, apigenin, and acacetin was prepared in methanol at 100 ng/mL. To evaluate linearity, mixed standard working solutions were prepared at concentrations of 0.1, 0.2, 0.5, 1.0, 5.0, 10, and 100 ng/mL. For sample preparation, approximately 35.66 mg of MPSK was transferred into a 1.5 mL microcentrifuge tube, and 1 mL of DMSO was added. To prepare a 5000 ppm stock solution, 140 µL of the DMSO extract was mixed with 860 µL of methanol. This stock was then diluted with methanol to a final concentration of 1000 ppm for HPLC analysis. Acacetin (00017), and apigenin (10798) were obtained from Sigma-Aldrich (St. Louis, MO, United States). Apigenin 7-glucoside (HY-N0578) and eugenin (HY33351) were purchased from MedChemExpress (NJ, United States).

Chromatographic separation was performed on a Phenomenex Kinetex C18 column (150 × 2.1 mm, 2.6 µm). The mobile phases were: (A) 0.1% formic acid in water and (B) 0.1% formic acid in acetonitrile. Gradient elution was carried out at a flow rate of 0.2 mL/min as follows: 0–15 min, 10%–90% B; 15–20 min, 90% B; 20–20.1 min, 90%–10% B; 20.1–25 min, 10% B. The column oven was maintained at 40 °C, the autosampler was set to 4 °C, and the injection volume was 5 µL. The total run time was 25 min. MS source parameters were set as follows: drying gas temperature, 200 °C; drying gas flow rate, 13 L/min; sheath gas temperature, 250 °C; sheath gas flow rate, 11 L/min; capillary voltage, 3000 V; and nozzle voltage, 1500 V.

The standards were analyzed in scan mode by selecting the most abundant ion as the precursor ion. Product ions were generated at various collision energies using product ion scan mode. For quantification, the most abundant, non-interfering fragment ion was used as the quantitative ion, accompanied by the next two most abundant fragment ions for qualitative confirmation.

### 2.4 Cell viability assay

RAW 264.7 macrophages or HEK 293 cells were seeded at a density of 5 × 10^4^ cells/well in 96-well plates and incubated overnight at 37 °C. Cells were pre-treated with MPSK (0, 10, 50, and 100 μg/mL) or vehicle control (DMSO) for 2 h. They were then stimulated with either LPS (1 μg/mL) or PMA; 50 nM for an additional 22 h at 37 °C. DMSO, LPS (L6529), and phorbol-12-myristate-13-acetate (PMA; P8139) were obtained from Sigma-Aldrich (St. Louis, MO, United States). Cell viability was assessed using the EZ-Cytox Cell Viability Assay Kit (DoGenBio, Seoul, Korea). The reagent was added at a 1:20 ratio of the culture volume, and cells were incubated for 30 min at 37 °C. Absorbance was measured at 450 nm using a Synergy H1 microplate reader (BioTek Instruments, Inc., Vermont, United States), with background absorbance at 650 nm subtracted from the final readings.

### 2.5 NO production assay

RAW 264.7 macrophages were seeded at a density of 5 × 10^4^ cells/well in 96-well plates and incubated overnight at 37 °C. Cells were pre-treated with MPSK (0, 10, 50, and 100 μg/mL) for 2 h, followed by stimulation with or without LPS (1 μg/mL) for 22 h at 37 °C. As a positive control, the methanol extract of *Mikania cordata* (Burm. f.) B. L. Rob. Leaves (MMC) was used at a concentration of 200 μg/mL, and DMSO was used as a vehicle control. To measure NO production, culture supernatants were transferred to a new 96-well plate, and Griess reagent (0.1% N-(1-naphthyl)ethylenediamine, 2.5% phosphoric acid (H_3_PO_4_), and 1% sulfanilamide in distilled water) was added. After 10 min of incubation at RT, absorbance was measured at 540 nm using a Synergy H1 microplate reader. NO concentrations were calculated using a sodium nitrite standard curve.

### 2.6 PGE_2_ production assay

RAW 264.7 macrophages were seeded in 96-well plates at 5 × 10^4^ cells/well and incubated overnight at 37 °C. After a 2 h pre-treatment with MPSK (0, 10, 50, and 100 μg/mL), cells were stimulated with LPS (1 μg/mL) for 22 h. Culture supernatants were collected by centrifugation at 3,000 rpm for 1 min at RT. PGE_2_ levels were quantified using the Prostaglandin E_2_ Express ELISA Kit (Cat. No. 500141, Cayman Chemical, MI, United States), following the manufacturer’s instructions. Briefly, the culture supernatant, PGE_2_-specific antibody, and tracer were added to a goat polyclonal anti-mouse pre-coated plate and incubated for 60 min. Ellman’s reagent was then added, and the plate was incubated in the dark for 90 min on an orbital shaker. Absorbance was measured at 420 nm using a Synergy H1 microplate reader.

### 2.7 Cytokine production assay

RAW 264.7 macrophage culture supernatants for cytokine quantification (IL-1β, IL-6, and TNF-α) were collected following the same procedure described for the PGE_2_ production assay. Cytokine concentrations were measured using Mouse Uncoated ELISA Kits (IL-1β: 88-7013A-88; IL-6: 88-7064-88; TNF-α: 88-7324-88; Thermo Fisher Scientific, Inc., Carlsbad, CA, United States), following the manufacturer’s instructions for a standard sandwich ELISA. Briefly, 96-well ELISA plates pre-coated with capture antibodies were blocked for 1 h at RT. Culture supernatants were then added and incubated for 2 h at RT, followed by incubation with biotin-conjugated detection antibodies and HRP-streptavidin. After 10 min of color development using TMB substrate, the reaction was stopped by adding 1 M phosphoric acid (H_3_PO_4_). Absorbance was measured at 450 nm using a Synergy H1 microplate reader. Cytokine concentrations were calculated from standard curves generated using the corresponding recombinant cytokine standards.

### 2.8 Immunoblot analysis

RAW 264.7 macrophages were treated with MPSK (0, 10, 50, and 100 μg/mL) or vehicle control (DMSO) for 2 h at 37 °C, followed by stimulation with or without LPS (1 μg/mL) at 37 °C. The duration of LPS treatment varied depending on the target protein: 3 min for IKKα/β, IκBα, NF-κB, and TAK1; 15 min for MAPKs (ERK, JNK, and p38); and 24 h for iNOS and COX-2. For the detection of Nrf2, Keap1, HO-1, and NQO1, cells were treated with MPSK alone (0, 10, 50, and100 μg/mL) for 24 h at 37 °C. Cells were lysed in ice-cold lysis buffer containing 20 mM Tris-HCl (pH 8.0), 150 mM NaCl, 0.5% Triton X-100, 1% glycerol, 1 mM EDTA, 2 mM phenylmethanesulfonyl fluoride, 10 mM NaF, and 1 mM Na_3_VO_4_. Lysates were centrifuged at 13,000 rpm for 20 min at 4 °C, and protein concentrations were determined using the Bradford assay (Bio-Rad, CA, United States) according to the manufacturer’s instructions. Samples were mixed with 5× SDS-PAGE loading buffer (250 mM Tris-HCl, pH 6.8; 10% SDS; 50% glycerol; 5% β-mercaptoethanol; 0.01% bromophenol blue) and boiled at 100 °C for 5 min. Equal amounts of protein were separated by 10% or 12% SDS-PAGE and transferred onto nitrocellulose membranes (GE Healthcare, WI, United States). Membranes were blocked in Tris-buffered saline with 0.05% Tween 20 (TBST) containing 5% nonfat dry milk for 1 h at RT, then incubated overnight at 4 °C with primary antibodies (1:1000 dilution in TBST with 5% Bovine serum albumin; BSA). The following day, membranes were incubated with HRP-conjugated secondary antibodies (1:5000 dilution in 5% nonfat dry milk in TBST) for 1 h at RT. Protein bands were visualized using enhanced chemiluminescence detection reagent (GBE-P100, DYNEBIO, Gyeonggi-do, Korea) and analyzed using LabWorks software (UVP Inc., CA, United States). The following antibodies were used in this study: anti-iNOS (sc-7271), anti-COX-2 (sc-1745), anti-JNK (sc-7345), anti-phospho-IκBα (Ser32/36; sc-8404), anti-IκBα (sc-1643), NF-κB p65 (sc-8008), IKKβ (sc-8014), anti-p38 (sc-7972), and HO-1 (sc-390991) from Santa Cruz Biotechnology, Inc. (CA, United States); anti-phospho-p38 (Thr180/Tyr182; #9211), anti-phospho-JNK (Thr183/Tyr185; #9252), anti-phospho-ERK1/2 (Thr202/Tyr204; #9106), anti-TAK1 (#4505), anti-phospho-TAK1 (Thr184/187; #4508), anti-phospho-IKKα/β (Ser176/180; #2697), anti-phospho-NF-κB p65 (Ser536; #3033), anti-NQO1 (#62262), anti-Keap1 (#8047), and anti-Nrf2 (#12721) from Cell Signaling Technology. Inc. (MA, United States); HRP-conjugated polyclonal goat anti-mouse IgG (GTX213111-01) and anti-rabbit IgG (GTX213110-01) from GeneTex Inc. (CA, United States).

### 2.9 Luciferase reporter assay

For reporter vector transfection, HEK 293 cells were seeded in 12-well plates at approximately 70% confluency and incubated overnight at 37 °C. Transfection was performed using polyethylenimine (PEI) with the reporter plasmids pNF-κB-Luc, pAP-1-Luc, or NQO1/ARE-Luc, along with the gWIZ-GFP plasmid as an internal control for normalization. After transfection, cells were cultured overnight at 37 °C. The following day, cells were pre-treated with MPSK (100 μg/mL) or positive control (MMC, 200 μg/mL) for 2 h, followed by stimulation with PMA and incubation for an additional 22 h at 37 °C. Cells were then lysed using Cell Culture Lysis Reagent (Promega, Madison, United States), and luciferase activity was measured using Luciferase Assay Reagent (Promega). Luminescence and GFP fluorescence were detected using a Synergy H1 microplate reader.

### 2.10 2,2-Diphenyl-1-picrylhydrazyl (DPPH) radical scavenging assay

To evaluate free radical scavenging activity, MPSK was dissolved in 100% methanol at final concentrations of 0, 50, 100, 200, and 500 μg/mL. Equal volumes of MPSK and 0.1 mM DPPH solution were mixed in a 96-well plate at a 1:1 ratio and incubated in the dark at 37 °C for 30 min. Quercetin (Q4951, Sigma-Aldrich) served as a positive control. Absorbance was measured at 517 nm using a Synergy H1 microplate reader. DPPH radical scavenging activity was calculated using the following formula:

$$\text{Scavenging activity }\left(\%\right)=\left[\left({\text{Abs}}_{\text{sample}}\;–\;{\text{Abs}}_{\text{blank}}\right)\;/\;{\text{Abs}}_{\text{blank}}\right]\times 100$$

where Abs_sample_ is the absorbance of the test sample and Abs_blank_ is the absorbance of methanol without DPPH.

### 2.11 Reverse transcription-quantitative polymerase chain reaction (RT-qPCR)

RAW 264.7 macrophages were seeded at a density of 2.5 × 10^5^ cells/well in 12-well plates and incubated overnight at 37 °C. Cells were then pre-treated with MPSK (0, 10, 50, and 100 μg/mL) for 2 h, followed by stimulation with lipopolysaccharide (LPS, 1 μg/mL). For the analysis of iNOS, COX-2, TNF-α, IL-6, and IL-1β, cells were harvested 3 h after LPS stimulation; for HO-1 and NQO1, cells were harvested after 24 h. Total RNA was extracted using Labozol reagent (Cosmogentech, Seoul, Korea) according to the manufacturer’s instructions. Chloroform was added, and samples were centrifuged at 13,000 rpm for 15 min. The aqueous phase was collected, mixed with isopropanol, gently inverted, and centrifuged again at 13,000 rpm for 10 min. The RNA pellet was then washed with 75% ethanol, centrifuged at 13,000 rpm for 5 min, air-dried, and dissolved in diethylpyrocarbonate (DEPC)-treated water. RNA concentration and purity were assessed using a NanoDrop ND-1000 spectrophotometer (NanoDrop Technologies, Rockland, United States). First-strand cDNA was synthesized using the TOPscript™ cDNA Synthesis Kit (Enzynomics, Daejeon, Korea) following the manufacturer’s instructions. Quantitative real-time PCR was performed using Dyne™ qPCR 2X Premix (DYNEBIO, Gyeonggi-do, Korea) and a CFX Connect Real-Time PCR System (Bio-Rad, Hercules, CA, United States). The thermal cycling conditions were as follows: initial denaturation at 95 °C for 3 min; 39 cycles of denaturation at 95 °C for 10 s, annealing at 55 °C for 20 s, and extension at 72 °C for 30 s. SYBR Green fluorescence was used to detect amplification, and expression levels were normalized to glyceraldehyde 3-phosphate dehydrogenase (GAPDH). Relative gene expression was calculated using the 2^–ΔΔCq^ method, with the control group set as 100%. Primer sequences used in this study are listed in Supplementary Table S1.

### 2.12 Immunofluorescence analysis of NF-κB nuclear translocation

To assess NF-κB nuclear translocation, RAW 264.7 macrophages were seeded at a density of 2 × 10^4^ cells/well in confocal dishes and cultured overnight at 37 °C. The cells were pre-treated with MPSK (100 μg/mL) for 2 h, followed by stimulation with LPS (1 μg/mL) for 3 min. After treatment, cells were washed three times with PBS and fixed with 4% formaldehyde for 15 min at 4 °C. The fixed cells were washed again with PBS and blocked with PBS containing 2% BSA for 1 h at RT. Next, the cells were incubated overnight at 4 °C with a primary antibody against NF-κB (1:100 dilution in 2% BSA). The following day, cells were incubated with an Alexa Fluor^®^ 594-conjugated goat anti-rabbit secondary antibody (1:500 dilution in 2% BSA) for 1 h at RT. After washing with PBS, the nuclei were counterstained with 1 μg/mL 4′,6-diamidino-2-phenylindole (DAPI) for 3 min. Immunofluorescence images were acquired using a Zeiss Laser Scanning Confocal Microscope (Carl Zeiss Microscopy, Thornwood, NY, United States).

### 2.13 Measurement of ROS generation

RAW 264.7 macrophages (3 × 10^5^ cells/well) were seeded in 12-well plates and incubated overnight at 37 °C in a humidified atmosphere. Cells were then treated with MPSK (0, 10, 50, and 100 μg/mL) or tert-Butylhydroquinone (tBHQ) (200 μM, used as a positive control) for 24 h at 37 °C. Following treatment, cells were washed twice with phosphate-buffered saline (PBS; pH 7.4) and incubated with 10 μM 2′,7′-dichlorofluorescein diacetate (DCF-DA) for 30 min at 37 °C in the dark. The DCF-DA solution was then removed, and the cells were gently washed twice with PBS. For ROS measurement, cells were lysed with lysis buffer containing 150 mM NaCl, 20 mM Tris-HCl (pH 8.0), 0.5% IGEPAL CA-630, 0.5% Triton X-100, 0.1 mM EDTA, and 1% glycerol. The lysates were centrifuged at 13,000 rpm for 15 min at 4 °C, and the supernatants were transferred to a black 96-well plate. Distilled water was used as a blank (n = 3). Fluorescence intensity was measured using a Synergy H1 microplate reader at excitation and emission wavelengths of 485 nm and 528 nm, respectively.

### 2.14 Statistical analysis

All numerical data are presented as the mean ± standard deviation (SD) from three independent experiments performed in triplicate. Statistical comparisons between groups were conducted using one-way ANOVA followed by the Holm–Šídák *post hoc* test in GraphPad Prism 8.0 software (GraphPad Software, CA, United States). Statistical significance was defined as follows: **p < 0.01, and ***p < 0.001 versus the non-treated control group; ^*a*^ *p* < 0.05, ^*b*^ *p* < 0.01, and ^*c*^ *p* < 0.001 versus the LPS-treated group. Non-significant differences were indicated as “n.s.” in the figures.

## 3 Results

### 3.1 Cell viability and morphological changes of MPSK in RAW 264.7 macrophages

**FIGURE 1:**
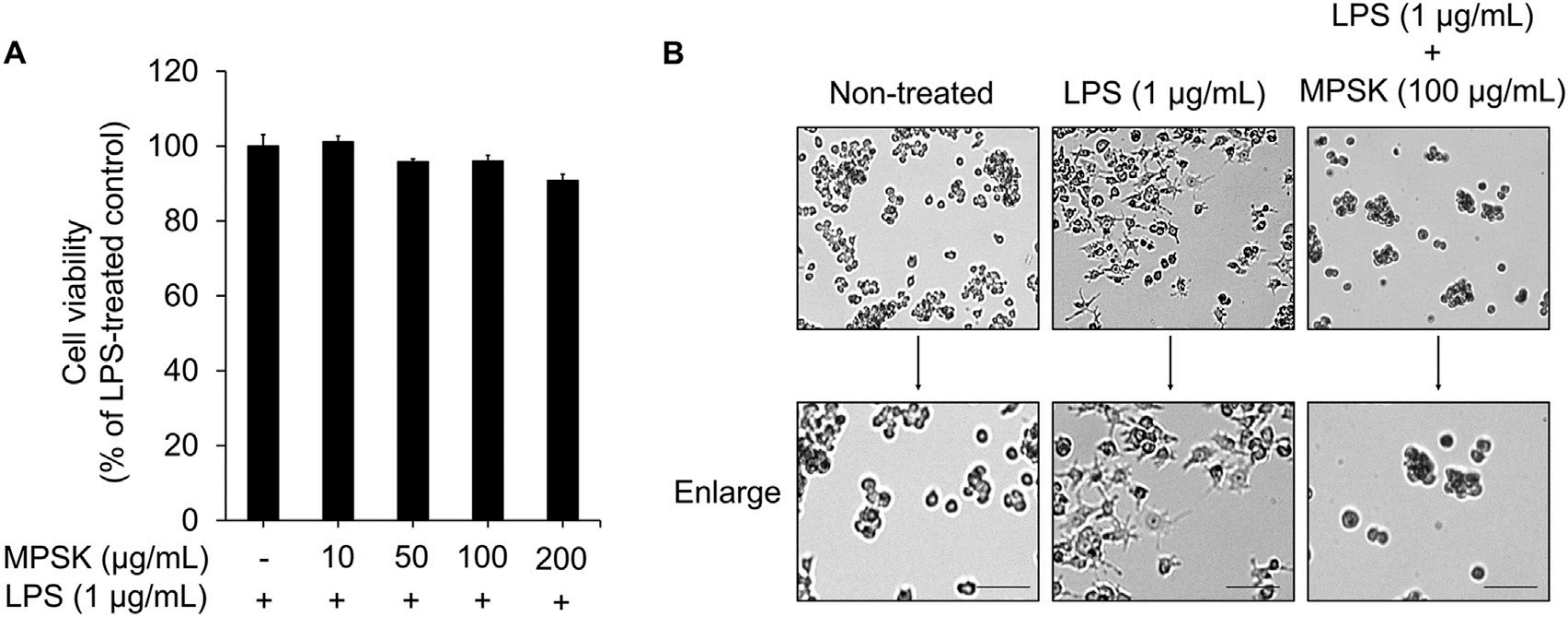
Effects of MPSK on cell viability and morphological changes in LPS-stimulated RAW 264.7 macrophages. RAW 264.7 macrophages were pretreated with various concentrations of MPSK (0, 10, 50, 100, or 200 μg/mL) for 2 h, followed by stimulation with LPS (1 μg/mL) for 22 h. **(A)** Cell viability was assessed using the EZ-Cytox assay kit. The bar graph shows the percentage of viable cells relative to the LPS-only group. Data are presented as the mean ± SD from three independent experiments. **(B)** Representative images of macrophage morphology were captured using an optical microscope at ×10 magnification. Scale bar = 50 μm.

To investigate the anti-inflammatory effects of MPSK, we first evaluated its cytotoxicity in RAW 264.7 macrophages. As shown in Figure 1A, MPSK exhibited no cytotoxic effects on RAW 264.7 cells, regardless of LPS stimulation. Based on these findings, 100 μg/mL was selected as the highest concentration for subsequent experiments assessing the anti-inflammatory activity of MPSK.

The morphology of RAW 264.7 cells was observed under a light microscope following LPS stimulation, either alone or in combination with MPSK, to examine morphological changes associated with inflammation and the potential modulatory effects of MPSK (Figure 1B). Unstimulated RAW 264.7 cells exhibited a typical spherical morphology. In contrast, LPS-treated cells showed marked morphological alterations, including the formation of long, thin dendritic-like projections, indicative of macrophage activation. Pre-treatment with MPSK for 2 h effectively suppressed these LPS-induced morphological changes, maintaining a rounded morphology similar to that of unstimulated cells. These results suggest that MPSK inhibits LPS-induced morphological alterations in RAW 264.7 macrophages.

Previous studies have reported that *P. stellatus* (Lour.) Kuntze contains bioactive compounds with anti-inflammatory properties (Gautam et al., 2010). The bioactive constituents isolated from *P. stellatus* (Lour.) Kuntze include flavonoids such as apigenin, acacetin, and luteolin, as well as eugenin and 5-hydroxy-6,7-dimethoxy-2-methylchromone (stellatin), which features a chromone skeleton (Gautam et al., 2010; Gautam et al., 2011). In the present study, five bioactive components (eugenin, luteolin, apigenin-7-glucoside, apigenin, and acacetin) were detected in MPSK using LC-MS/MS analysis (Supplementary Figure S1). These compounds were identified by comparing their retention times and accurate masses with those of authentic standards. The quantified contents of these compounds in MPSK were as follows: eugenin (44.04 ng/mg), luteolin (22.69 ng/mg), apigenin-7-glucoside (16.48 ng/mg), apigenin (5.97 ng/mg), and acacetin (0.83 ng/mg).

### 3.2 Inhibitory effect of MPSK on NO and PGE_2_ production by regulating iNOS and COX-2 expression in LPS-induced RAW 264.7 macrophages

**FIGURE 2:**
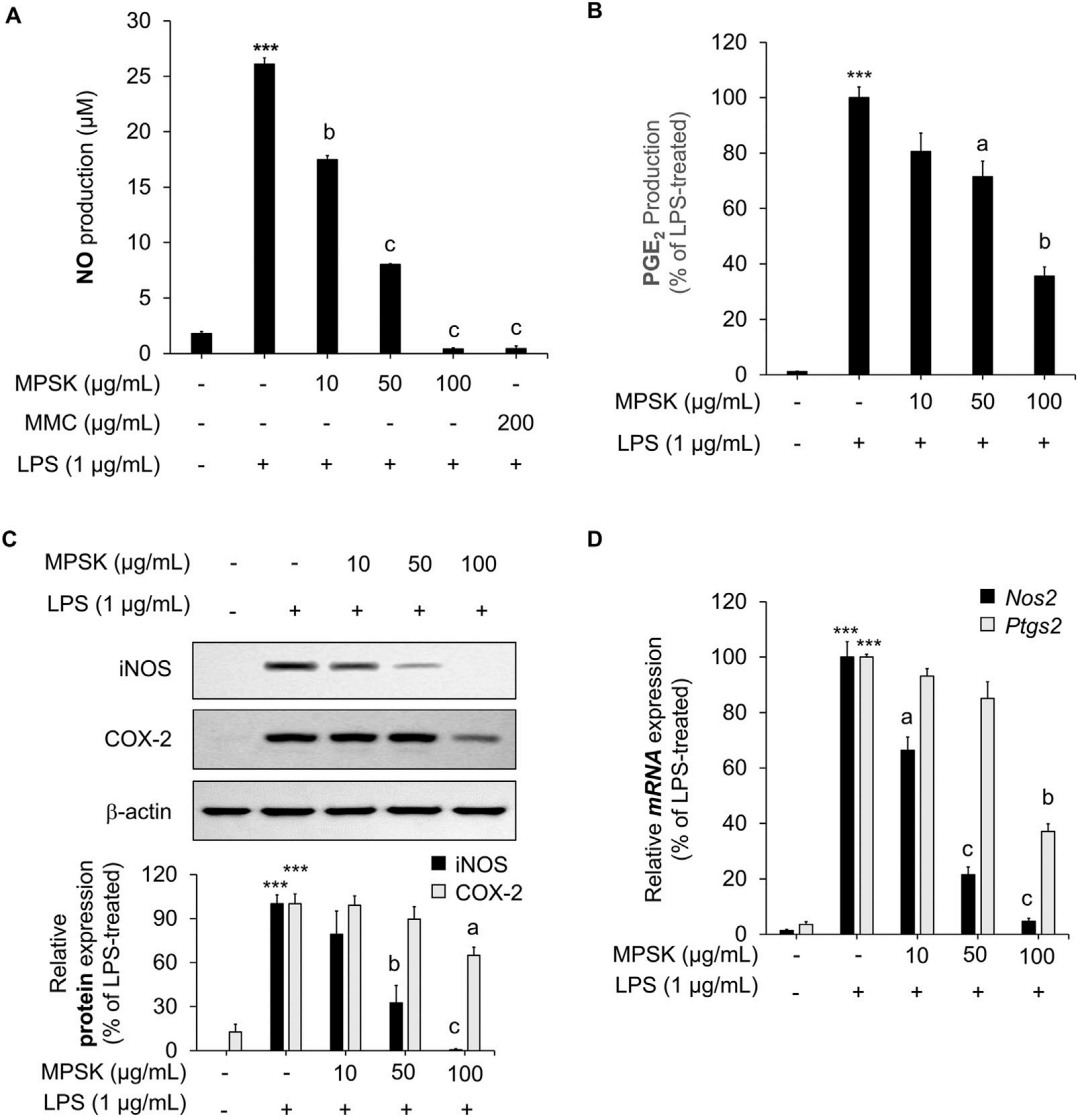
Effect of MPSK on the expression of inflammatory mediators in LPS-stimulated RAW 264.7 macrophages. **(A–C)** RAW 264.7 macrophages were pretreated with MPSK (0, 10, 50, and 100 μg/mL) for 2 h, followed by stimulation with LPS (1 μg/mL) for 22 h **(A)** NO levels in the culture supernatant were measured using the Griess reagent. MMC was used as a positive control. Results were calculated based on a nitrite standard curve and normalized to the LPS-only group. **(B)** PGE_2_ production was quantified by ELISA. **(C)** Protein expression levels of iNOS and COX-2 were analyzed by immunoblotting, with β-actin used as a loading control. **(D)** For mRNA analysis, cells were pretreated with MPSK for 2 h and stimulated with LPS for 3 h. The mRNA expression of *Nos2* and *Ptgs2* was determined by RT-qPCR and normalized to *Gapdh*. All experiments were independently performed in triplicate. Data are presented as the mean ± SD. ^***^ *p* < 0.001 vs. untreated control; ^a^ *p* < 0.05, ^b^ *p* < 0.01, ^c^ *p* < 0.001 vs. LPS-treated group.

To evaluate the inhibitory effects of MPSK on inflammatory mediator production, RAW 264.7 cells were pre-treated with various concentrations of MPSK for 2 h, followed by LPS stimulation. LPS treatment significantly increased the production of NO and PGE_2_ in RAW 264.7 cells, while MPSK co-treatment markedly reduced the levels of these inflammatory mediators in a dose-dependent manner (Figures 2A,B). MMC, a positive control used in the NO assay, has been reported to be a natural compound that exhibits anti-inflammatory effects by regulating the NF-κB and MAPK pathways. To determine whether the bioactive compounds identified in MPSK (eugenin, luteolin, apigenin-7-glucoside, apigenin, and acacetin) contributed to NO inhibition, each compound was evaluated individually at concentrations equivalent to their respective amounts in 100 μg/mL of MPSK. Notably, these individual compounds exhibited only limited inhibitory effects on NO production compared to the whole MPSK extract (Supplementary Figure S2). As none of the tested compounds induced cytotoxicity, the limited NO suppression suggests that the anti-inflammatory effects of MPSK are not solely attributable to these individual components. Instead, the observed effects likely result from additional unidentified bioactive constituents or synergistic interactions among the components in MPSK.

Given that NO and PGE_2_ production are mediated by iNOS and COX-2, respectively, we further investigated whether MPSK regulates the expression of these enzymes. LPS stimulation significantly upregulated the protein expression of iNOS and COX-2 in RAW 264.7 cells, whereas MPSK treatment suppressed their expression in a dose-dependent manner (Figure 2C). A similar trend was observed at the mRNA level: MPSK treatment led to reduced *Nos2* (iNOS encoded by the *Nos2* gene) and *Ptgs2* (COX-2 encoded by the *Ptgs2* gene) mRNA expression, consistent with the observed decrease in protein levels (Figure 2D). Notably, at 50 μg/mL, MPSK exerted a stronger inhibitory effect on *Nos2* expression compared to *PtgsS2*, which aligned with the more pronounced reduction in NO than in PGE_2_ levels. Collectively, MPSK suppresses the production of the inflammatory mediators NO and PGE_2_ by downregulating the expression of iNOS and COX-2 at both the transcriptional and translational levels in LPS-stimulated RAW 264.7 cells. These findings indicate that MPSK particularly exerts a more potent regulatory effect on iNOS.

### 3.3 Attenuating effect of MPSK on inflammatory cytokine expression in LPS-induced RAW 264.7 macrophages

**FIGURE 3:**
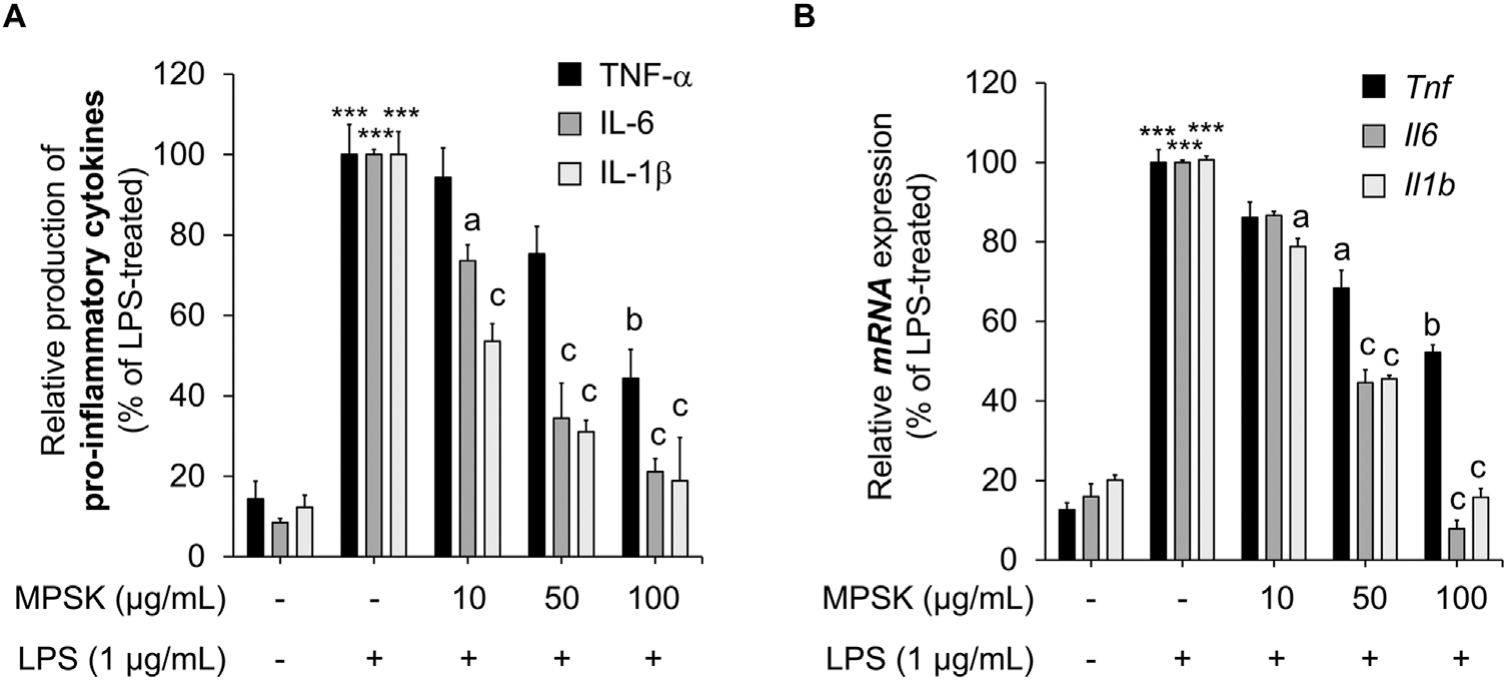
Effect of MPSK on the production and mRNA expression of inflammatory cytokines in LPS-stimulated RAW 264.7 macrophages. RAW 264.7 macrophages were pretreated with MPSK (0, 10, 50, and 100 μg/mL) for 2 h, followed by stimulation with LPS (1 μg/mL). **(A)** After 3 h of LPS treatment, the levels of secreted inflammatory cytokines IL-6, IL-1β, and TNF-α in the culture supernatant were measured using ELISA kits. **(B)** mRNA expression levels of *Il6*, *Il1b*, and *Tnf* were analyzed by RT-qPCR after 3 h of LPS stimulation. Gene expression was normalized to *Gapdh* and expressed relative to the LPS-treated control group. Data represent the mean ± SD of three independent experiments. ^***^ *p* < 0.001 vs. untreated control; ^a^ *p* < 0.05, ^b^ *p* < 0.01, ^c^ *p* < 0.001 vs. LPS-treated group.

Upon LPS stimulation, RAW 264.7 macrophages, which are well-known for their preserved immune functionality, activate inflammatory signaling pathways and induce the expression of key pro-inflammatory cytokines, including TNF-α, IL-1β, and IL-6 (Zhang and An, 2007; M. Kim et al., 2024). To further investigate the anti-inflammatory mechanism of MPSK, we assessed its effects on both mRNA and protein expression levels of these cytokines in LPS-stimulated RAW 264.7 cells. LPS treatment markedly increased the protein levels of TNF-α, IL-1β, and IL-6 compared to untreated controls (Figure 3A). Pre-treatment with MPSK significantly and dose-dependently reduced the protein expression of all three cytokines. Consistent with these results, MPSK also suppressed the mRNA expression of *Tnf*, *Il1b*, and *Il6* genes in a concentration-dependent manner (Figure 3B). Although MPSK effectively reduced both mRNA and protein levels of these pro-inflammatory cytokines, the extent of TNF-α suppression was relatively modest compared to IL-1β and IL-6, suggesting differential sensitivity of cytokine regulation to MPSK treatment.

### 3.4 Inhibitory effect of MPSK on NF-κB and AP-1 transcriptional activation in HEK 293 cells

**FIGURE 4:**
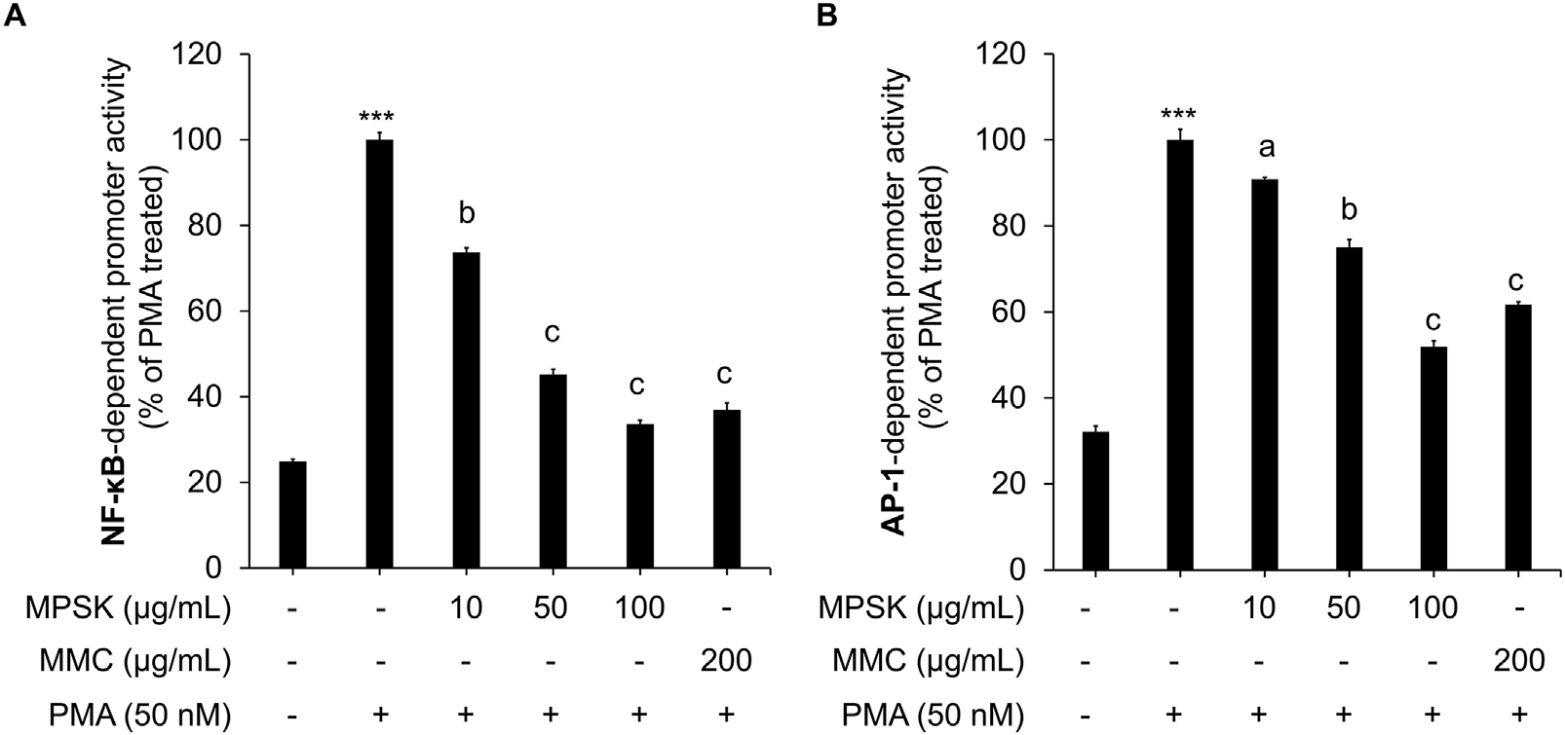
Transcriptional regulation of NF-κB and AP-1 by MPSK. To evaluate the effects of MPSK on transcription factor activity, HEK 293 cells were transfected with luciferase reporter constructs containing binding sites for **(A)** NF-κB or **(B)** AP-1, along with the gWIZ-GFP plasmid as an internal control. Following transfection, cells were pretreated with various concentrations of MPSK (0, 10, 50, and 100 μg/mL) or MMC (200 μg/mL; positive control) for 2 h, then stimulated with PMA (50 nM) for 22 h. Luciferase activity was normalized to GFP fluorescence intensity. Data are presented as the mean ± SD of three independent experiments. ^***^ *p* < 0.01 vs. untreated control; ^a^ *p* < 0.05, ^b^ *p* < 0.01, ^c^ *p* < 0.001 vs. PMA-treated group.

To elucidate the molecular mechanisms by which MPSK modulates cytokine production and inflammatory mediator expression, we examined its effects on the transcriptional activity of NF-κB and AP-1, which are critical downstream effectors of the NF-κB and MAPK signaling pathways, respectively. These pathways are typically activated by LPS through TLR4 stimulation by LPS (M. Kim et al., 2024). In this study, HEK 293 cells were transfected with luciferase reporter constructs containing either NF-κB or AP-1-responsive promoters to quantitatively assess transcriptional activation. Due to the low endogenous expression of TLR4 in HEK 293 cells, PMA was used instead of LPS to induce activation of these transcription factors, as PMA effectively and directly activates both NF-κB and AP-1. MPSK treatment significantly suppressed PMA-induced increase in luciferase reporter activity, indicating an inhibitory effect on NF-κB and AP-1 reporter assays (Figures 4A,B), indicating that MPSK inhibits the transcriptional activation of these key inflammatory mediators. Notably, NF-κB activity was more sensitive to MPSK inhibition than AP-1, with significant suppression observed even at lower concentrations. These findings suggest that the anti-inflammatory effects of MPSK are primarily mediated through the potent inhibition of NF-κB activation, with lesser but still notable effects on AP-1. Importantly, MPSK exerted these effects without inducing cytotoxicity in HEK 293 cells (Supplementary Figure S3).

### 3.5 Inhibition of MAPK and NF-κB signaling pathways by MPSK in LPS-stimulated RAW 264.7 macrophages

**FIGURE 5:**
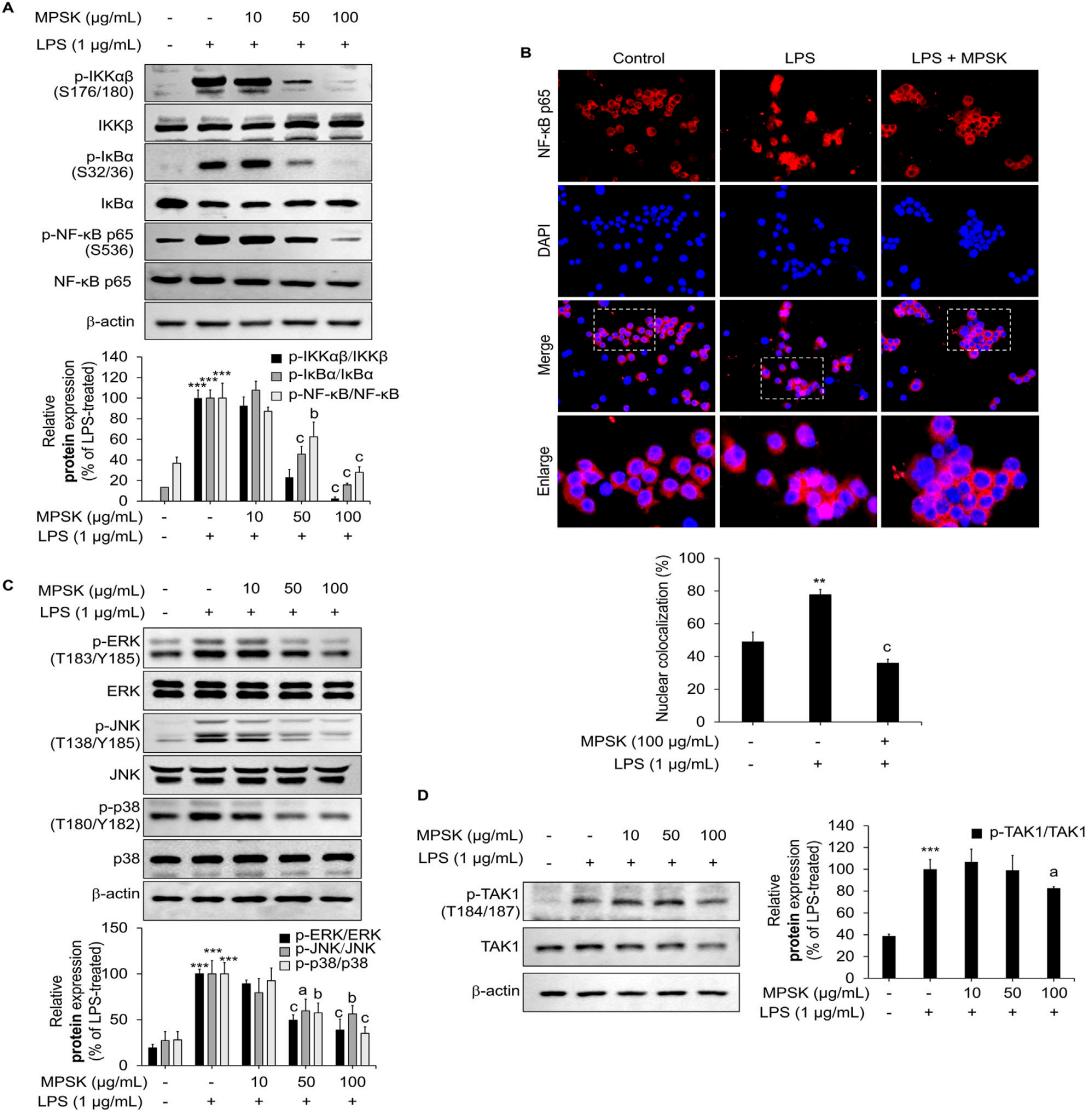
Effect of MPSK on NF-κB and MAPK signaling pathways in LPS-stimulated RAW 264.7 macrophages. RAW 264.7 cells were pretreated with MPSK (0, 10, 50, and100 μg/mL) for 2 h, followed by stimulation with LPS (1 μg/mL). **(A)** To evaluate NF-κB pathway activation, cells were stimulated with LPS for 3 min, and protein levels were analyzed by immunoblotting. **(B)** Nuclear translocation of the NF-κB p65 subunit was assessed by immunofluorescence using an anti-NF-κB antibody and Alexa Fluor 594-conjugated secondary antibody. Nuclei were counterstained with DAPI, and images were captured using a confocal microscope. NF-κB p65 nuclear localization was quantified using ImageJ software. Colocalization (%) was determined by calculating the ratio of the red fluorescent protein-positive nuclear area to the total DAPI-stained area. **(C)** To examine MAPK pathway activation, cells were stimulated with LPS for 15 min, and phosphorylation levels of ERK, JNK, and p38 were analyzed by immunoblotting. **(D)** TAK1 phosphorylation was also assessed via immunoblotting after 15 min of LPS stimulation. Phosphorylated protein levels were normalized to total protein levels and quantified as bar graphs. Data are presented as the mean ± SD from three independent experiments. ^***^ *p* < 0.01 vs. untreated control; ^a^ *p* < 0.05, ^b^ *p* < 0.01, ^c^ *p* < 0.001 vs. LPS-treated group.

To investigate whether MPSK regulates NF-κB and AP-1 transcriptional activity through upstream signaling cascades, we conducted immunoblot analyses to assess the activation status of key components in the NF-κB and MAPK pathways in LPS-stimulated RAW 264.7 cells. As shown in Figure 5A, pre-treatment with MPSK at concentrations of 50 μg/mL or higher dose-dependently suppressed the phosphorylation of IKKα/β, IκBα, and NF-κB p65. LPS stimulation significantly increased the phosphorylation of IκBα at Ser32/36, which was accompanied by a reduction in total IκBα protein levels, consistent with its degradation via the ubiquitin–proteasome pathway. MPSK pre-treatment effectively prevented this degradation, indicating inhibition of NF-κB activation. Moreover, MPSK reduced the phosphorylation of NF-κB p65 at Ser536, a modification known to enhance its transcriptional activity and promote nuclear translocation. Supporting these findings, confocal immunofluorescence microscopy revealed that MPSK significantly attenuated LPS-induced nuclear translocation of NF-κB (Figure 5B).

MPSK also modulated the MAPK signaling pathway, indicating a broader regulatory effect on inflammatory signaling (Figure 5C). LPS stimulation led to increased phosphorylation of JNK, p38, and ERK in RAW 264.7 cells, whereas MPSK pre-treatment attenuated the phosphorylation of all three MAPKs. Notably, this inhibitory effect on MAPK phosphorylation became more pronounced at 100 μg/mL, while suppression of NF-κB pathway activation was evident at lower concentrations. These results suggest that the NF-κB pathway is more sensitive to MPSK treatment than the MAPK pathway.

Given that TAK1 acts as a common upstream regulator of both NF-κB and MAPK signaling cascades (Park et al., 2024; Kang et al., 2023), we next examined whether MPSK targets TAK1 to exert its anti-inflammatory effects (Figure 5D). Immunoblot analysis revealed a marked increase in TAK1 phosphorylation upon LPS stimulation, consistent with its activation. However, MPSK pre-treatment did not significantly reduce TAK1 phosphorylation, suggesting that MPSK does not act directly on TAK1. Collectively, these results indicate that MPSK exerts its anti-inflammatory effects primarily by targeting downstream components of the NF-κB and MAPK signaling pathways, rather than upstream kinases such as TAK1.

### 3.6 Antioxidant effects of MPSK via Nrf2 signaling pathway activation in RAW 264.7 macrophages

**FIGURE 6:**
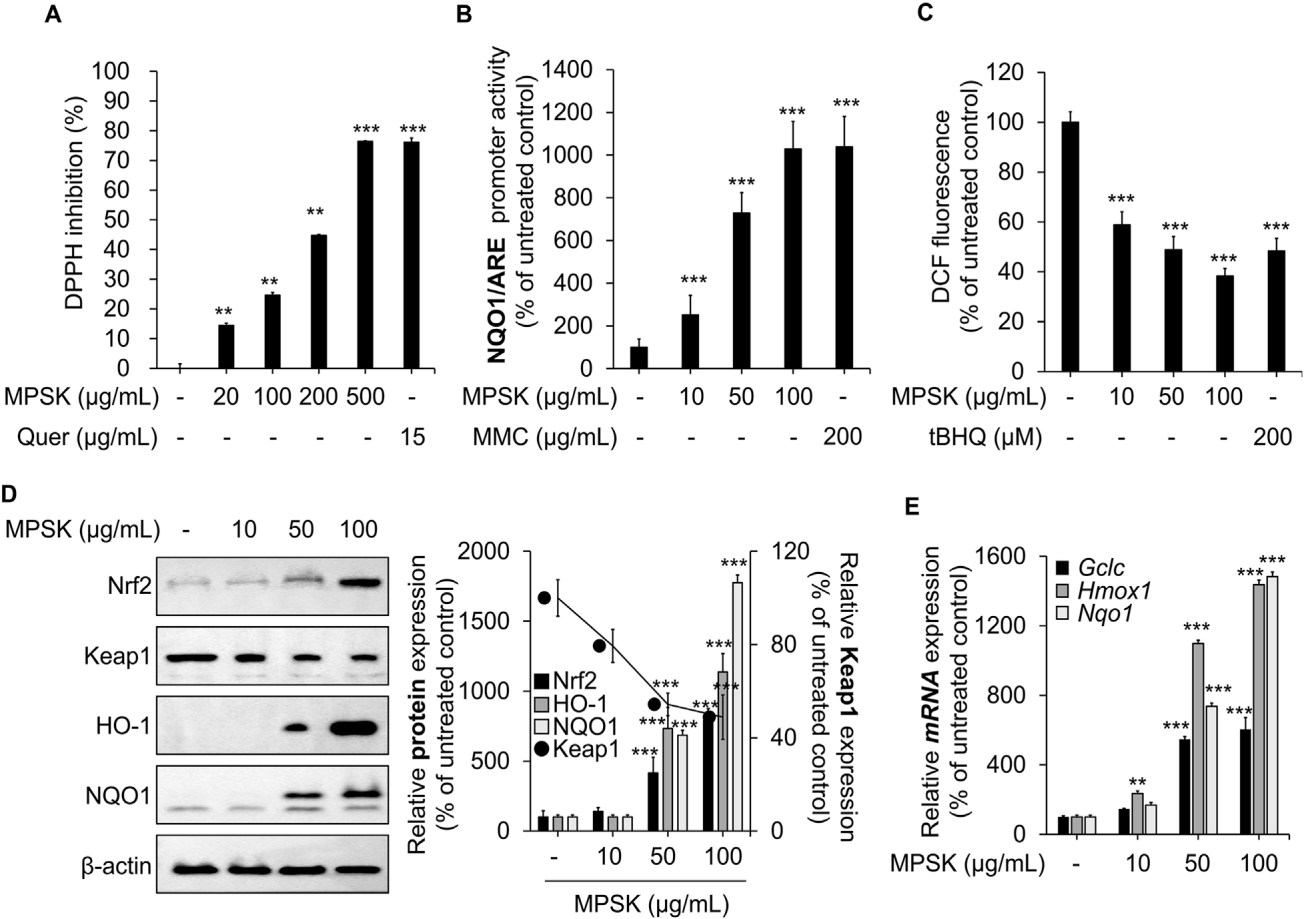
Modulation of Nrf2 and its downstream target genes by MPSK in RAW 264.7 macrophages. **(A)** The radical scavenging activity of MPSK was evaluated using the *in vitro* DPPH assay. Methanol served as a negative control (set at 0%), and quercetin (Quer) was used as a positive control. Data are presented as the mean ± SD from three independent experiments. **(B)** HEK 293 cells were transfected with the NQO1/ARE luciferase reporter plasmid and the gWIZ-GFP plasmid for normalization. Cells were treated with MPSK or MMC for 24 h, and luciferase activity was measured and normalized to GFP fluorescence. MMC was used as a positive control. **(C)** RAW 264.7 macrophages were treated with MPSK (10, 50, and 100 μg/mL) or tBHQ (200 μM; positive control) for 24 h. Intracellular ROS levels were measured using DCF-DA fluorescence (Ex/Em: 485/528 nm), and fluorescence intensity was quantified with a microplate reader. **(D)** Cells were treated with MPSK (100 μg/mL) for 24 h. Total protein lysates were analyzed by immunoblotting to assess the expression of Nrf2, Keap1, HO-1, and NQO1. **(E)** mRNA expression levels of *Gclc*, *Hmox1*, and *Nqo1* were analyzed by RT-qPCR following 24 h of MPSK treatment and normalized to *Gapdh*. All data are presented as the mean ± SD from three independent experiments. ^**^ *p* < 0.01, ^***^ *p* < 0.001 vs. untreated control.

Nrf2 is a key regulator of the cellular antioxidant response, playing a critical role in the clearance of ROS and the suppression of inflammation (Kang et al., 2023; Park et al., 2024). The antioxidant potential of MPSK was initially evaluated *in vitro* using the DPPH radical scavenging assay, a widely used method to assess free radical neutralizing activity (Figure 6A). Quercetin, a well-established antioxidant, served as a positive control. MPSK exhibited dose-dependent DPPH radical scavenging activity, with its effect at 500 μg/mL comparable to that of quercetin. To identify the specific compounds contributing to the antioxidant activity of MPSK, the DPPH scavenging capacities of its major bioactive components (eugenin, luteolin, apigenin-7-glucoside, apigenin, and acacetin) were evaluated individually (Supplementary Figure S4). Among these, luteolin displayed the highest radical scavenging activity, comparable to MPSK at 100 μg/mL. To further assess the cellular antioxidant activity of MPSK, we examined the transcriptional activity of the ARE in the NQO1 promoter using a luciferase reporter assay (Figure 6B). MPSK significantly enhanced ARE-driven promoter activity at concentrations of 50 μg/mL or higher. MMC, a natural extract known to activate Nrf2, was used as a positive control. MPSK enhanced ARE activity in a manner comparable to MMC, even at lower concentrations. As ARE is a cis-regulatory element in promoters of Nrf2-responsive genes, this finding suggests that MPSK activates the Nrf2 signaling pathway, likely by promoting Nrf2 nuclear translocation and transcriptional activation. To confirm Nrf2 pathway activation, intracellular ROS levels were measured in RAW 264.7 macrophages after MPSK treatment (Figure 6C). MPSK significantly reduced ROS accumulation in a dose-dependent manner, consistent with activation of the Nrf2-mediated antioxidant defense system. Given that tBHQ has been reported to enhance antioxidant activity by reducing intracellular ROS levels, it was served as a positive control. We next examined Nrf2 pathway activation at both the protein and transcriptional levels (Figure 6D). MPSK treatment increased Nrf2 protein levels while decreasing those of Keap1, a cytoplasmic inhibitor that targets Nrf2 for degradation. In line with this, MPSK dose-dependently upregulated the protein expression of Nrf2 downstream targets, including HO-1 and NQO1. Additionally, MPSK significantly increased the mRNA expression of key Nrf2-regulated genes such as glutamate-cysteine ligase catalytic subunit (*Gclc*), *Nqo1*, and *Hmox1*, a gene encoding HO-1 (Figure 6E). Collectively, these results show that MPSK exerts strong antioxidant effects by activating the Nrf2 signaling pathway, leading to the induction of key antioxidant enzymes and contributing to the maintenance of cellular redox homeostasis.

## 4 Discussion

The anti-inflammatory and antioxidant properties of plant-derived extracts have been widely studied. However, the molecular mechanisms underlying these effects in *P. stellatus* (Lour.) Kuntze, a member of the Lamiaceae family, remain poorly understood. In this study, we investigated the anti-inflammatory and antioxidant activities of MPSK in LPS-stimulated RAW 264.7 cells, with a particular focus on its regulatory effects on inflammatory signaling and oxidative stress.

Macrophages are central mediators of immune and inflammatory responses and are capable of undergoing functional reprogramming in response to environmental cues, a process known as macrophage polarization (C.-H. Lee and Choi, 2018). Classically activated (M1) polarization is typically induced by pro-inflammatory stimuli such as LPS, interferon-γ, and TNF-α, and is characterized by a robust pro-inflammatory phenotype. In RAW 264.7 macrophages, LPS-induced M1 polarization is often accompanied by pronounced morphological changes, including the formation of dendritic spine-like projections (Orecchioni et al., 2019; Lebaudy et al., 2021). Notably, treatment with MPSK effectively inhibited these LPS-induced morphological alterations. M1 polarization is also associated with increased expression of pro-inflammatory mediators, including TNF-α, IL-1β, IL-6, NO, and ROS (Lebaudy et al., 2021; Orecchioni et al., 2019). In our study, MPSK significantly downregulated both protein and mRNA levels of iNOS, a key marker of M1 macrophages, and markedly suppressed the production of IL-1β, IL-6, and TNF-α. These findings highlight the potential of MPSK to inhibit LPS-induced M1 polarization in RAW 264.7 macrophages and to attenuate the accompanying inflammatory responses.

Among the intracellular signaling pathways involved in regulating inflammatory mediators and cytokines, the NF-κB and MAPK pathways are particularly prominent (M. Kim et al., 2024; Wei et al., 2022). In this study, MPSK inhibited the transcriptional activities of both NF-κB and AP-1, with a more pronounced suppressive effect observed on NF-κB. Our previous research demonstrated that inhibition of NF-κB activity is primarily associated with the downregulation of iNOS, IL-6, and IL-1β, whereas suppression of AP-1 more directly affects the expression of TNF-α and COX-2 (Kang et al., 2023). Consistent with these observations, MPSK significantly reduced NF-κB activity, leading to decreased expression of iNOS, IL-6, and IL-1β.

The differential regulation of inflammatory mediators by NF-κB and AP-1 may depend on cell type–specific contexts and the nature of the activating stimuli, which influence promoter-binding specificity and the recruitment of transcriptional co-regulators. MPSK-mediated suppression of NF-κB and AP-1 transcriptional activity was linked to the inhibition of their upstream signaling cascades. Specifically, MPSK attenuated NF-κB signaling by inhibiting phosphorylation at Ser176/180 on IKKα/β, thereby reducing IκBα phosphorylation at Ser32/36 and subsequently suppressing phosphorylation of NF-κB p65 at Ser536, an essential step for its nuclear translocation. Additionally, MPSK inhibited the phosphorylation of MAPKs, including ERK, JNK, and p38. However, significant inhibition of MAPK signaling was observed only at the highest tested concentration of 100 μg/mL, suggesting that the MAPK pathway is less sensitive to MPSK treatment than the NF-κB pathway. Taken together, these findings suggest that the NF-κB pathway is more prominently affected by MPSK and may represent the principal target of its anti-inflammatory activity. The observation that TAK1, an upstream kinase commonly involved in both the NF-κB and MAPK pathways, was not clearly regulated by MPSK requires further investigation. This finding suggests that MPSK may exert its effects through alternative upstream regulators of these signaling pathways. While TAK1 is known to be a major mediator of LPS-induced signaling, LPS can also activate the Src family of tyrosine kinases as a secondary pathway (Maa et al., 2008). Src has been identified as a critical regulator of NF-κB and MAPK signaling in LPS-induced acute kidney injury (Li et al., 2022; H. S. Lee et al., 2007), and c-Src activation has been implicated in promoting Nrf2 nuclear translocation and transcriptional activity (Fão et al.,2019). Taken together, MPSK may exert its pharmacological effects by targeting Src, a key upstream regulator of NF-κB, MAPK, and Nrf2 signaling pathways.

The presence of five bioactive compounds (eugenin, luteolin, apigenin-7-glucoside, apigenin, and acacetin) in MPSK was confirmed through LC/MS-MS analysis. These naturally occurring molecules have well-documented anti-inflammatory activities and have been reported to suppress the production of key pro-inflammatory cytokines, including TNF-α, IL-1β, and IL-6 (Gautam et al., 2010; Aziz et al., 2018; Huang et al., 2013; Fuchs and Milbradt, 1993; Pan et al., 2006). Among them, luteolin and apigenin are particularly notable for their ability to modulate inflammatory and oxidative responses by activating the Nrf2 signaling pathway, leading to enhanced expression of antioxidant enzymes and reduced cytokine production in both LPS-stimulated RAW 264.7 cells and animal models (Lv et al., 2025; Jamshidi et al., 2021). However, quantitative profiling revealed that the concentrations of these compounds in MPSK were relatively low, present only at the ng/mg level. When tested at concentrations equivalent to their respective levels in 100 μg/mL of MPSK, none of the individual compounds replicated the NO-reducing effect observed with the whole extract. Furthermore, DPPH radical scavenging activity was primarily attributed to luteolin, while the other compounds exhibited limited or undetectable antioxidant activity *in vitro*. These findings suggest that the potent anti-inflammatory and antioxidant effects of MPSK cannot be solely attributed to its known constituents. Instead, they imply the potential involvement of additional, yet unidentified bioactive molecules or synergistic interactions among the extract’s diverse phytochemicals. Since the five identified compounds in MPSK did not exhibit anti-inflammatory activity at concentrations equivalent to those in MPSK, comprehensive phytochemical profiling is necessary to identify additional bioactive constituents. Further investigation of anti-inflammatory properties of additional bioactive constituents, alongside detailed elucidation of their molecular targets and signaling pathways, may provide critical insights into the mechanisms responsible for the anti-inflammatory and antioxidant activities of MPSK.

The Nrf2 signaling pathway plays a crucial role in cellular defense by mitigating oxidative stress and inflammatory responses, especially in the context of inflammatory diseases (Canton et al., 2021). In this study, MPSK promoted the proteasomal degradation of Keap1, a negative regulator of Nrf2, thereby enhancing Nrf2 stabilization and activation. This activation led to increased mRNA and protein expression of key downstream antioxidant genes, including *Gclc*, *Hmox1*, and *Nqo1*. Although MPSK demonstrated relatively weak radical scavenging activity in chemical assays, it exhibited marked antioxidant effects at the cellular level through Nrf2 pathway activation. These results highlight the discrepancy between *in vitro* radical scavenging assays and cellular antioxidant responses, underscoring the importance of Nrf2-mediated mechanisms in the antioxidant properties of MPSK.

To further verify the reliability and robustness of the anti-inflammatory and antioxidant effects of MPSK, *in vivo* studies are essential. In this study, we mechanistically confirmed the effects of MPSK in RAW 264.7 macrophages. However, *in vitro* efficacy alone is insufficient to assess physiological relevance. MPSK exhibited anti-inflammatory and antioxidant activity at a concentration of 200 μg/mL in cell-based assays. *In vivo* experiments are typically conducted using doses approximately 12–20 times higher than those used in cell culture studies. Therefore, future studies should take these concentrations into account when evaluating the efficacy of MPSK in animal models.

## 5 Conclusion

This study provides a comprehensive understanding of the molecular mechanisms underlying the anti-inflammatory and antioxidant activities of MPSK in LPS-stimulated RAW 264.7 macrophages. MPSK significantly suppressed inflammatory mediator and cytokine production by inhibiting M1 macrophage polarization and downregulating the NF-κB and MAPK signaling pathways. In parallel, MPSK activated the Nrf2 signaling pathway, resulting in the upregulation of key antioxidant genes, particularly HO-1. Taken together, these findings suggest that MPSK may serve as a promising candidate for the development of therapeutic agents targeting inflammation-related disorders.

## Data Availability

The original contributions presented in the study are included in the article/Supplementary Material, further inquiries can be directed to the corresponding author.

## Glossary

ANOVA: one-way analysis of variance
AP-1: activator protein 1
ARE: antioxidant response element
COX-2: cyclooxygenase-2
DCF-DA: 2′,7′-dichlorofluorescein diacetate
DPPH: 2,2-diphenyl-1-picrylhydrazyl
ELISA: enzyme-linked immunosorbent assay
ERK: extracellular signal-regulated kinase
GAPDH: glyceraldehyde 3-phosphate dehydrogenase
GCLC: glutamate-cysteine ligase catalytic subunit
GFP: green fluorescent protein
HO-1: heme oxygenase-1
IκB: inhibitor of NF-κB
IKK: IκB kinase
IL-1β: interleukin-1β
IL-6: interleukin-6
iNOS: inducible NO synthase
JNK: c-Jun N-terminal kinase
Keap1: Kelch-like ECH-associated protein 1
LPS: lipopolysaccharide
MAPK: mitogen-activated protein kinase
MPSK: methanol extract of *Pogostemon stellatus* (Lour.) Kuntze
NF-κB: nuclear factor-κB
MRM: multiple reaction monitoring
NO: nitric oxide
NQO1: NAD(P)H quinone oxidoreductase 1
Nrf2: nuclear factor erythroid-2-related factor 2
PBS: phosphate-buffered saline
PGE2: prostaglandin E2
PMA: phorbol 12-myristate 13-acetate
qRT-PCR: quantitative reverse transcription-polymerase chain reaction
ROS: reactive oxygen species
SD: standard deviation
TAK1: transforming growth factor-β-activated kinase 1
TLR4: toll-like receptor 4
TNF-α: tumor necrosis factor-α
TRAF6: TNF receptor-associated factor 6
