# Supplementary material for: Anti-inflammatory and antioxidant effects of Pogostemon stellatus (Lour.) Kuntze via MAPK, NF-κB, and Nrf2 signaling pathways in LPS-activated RAW 264.7 macrophages
Source: Front Pharmacol. 2025 Sep 25;16:1679919. doi: 10.3389/fphar.2025.1679919 (PMC12507890; doi:10.3389/fphar.2025.1679919)
Supplement: Supplementary file 1 [file DataSheet1.docx]

Supplementary Meterials

# Supplementary Table

- 1. **Supplementary Table S1. Primer sequences used in RT-qPCR.**

| **Primer name** | | **Sequence (5'–3')** |
| --- | --- | --- |
| ***Nos2*** | Forward | CGA AAC GCT TCA CTT CCA A |
|  | Reverse | TGA GCC TAT ATT GCT GTG GCT |
| ***Ptgs2*** | Forward | GAT GCT CTT CCG AGC TGT G |
|  | Reverse | GGA TTG GAA CAG CAA GGA TTT |
| ***Tnf*** | Forward | CTG TAG CCC ACG TCG TAG C |
|  | Reverse | TTG AGA TCC ATG CCG TTG |
| ***Il6*** | Forward | TCT AAT TCA TAT CTT CAA CCA AGA GG |
|  | Reverse | TGG TCC TTA GCC ACT CCT TC |
| ***Il1b*** | Forward | TTG ACG GAG CCC AAA AGA T |
|  | Reverse | GAT GTG CTG CTG CGA GAT T |
| ***Gapdh*** | Forward | GCT CTC TGC TCC TCC TGT TC |
|  | Reverse | ACG ACC AAA TCC GTT GAC TC |
| ***Gclc*** | Forward | GGA CAA ACC CCA ACC AT |
|  | Reverse | GTT GAA CTC AGA CAT CGT T |
| ***Hmox1*** | Forward | CCT TCC CGA ACA TCG ACA GCC |
|  | Reverse | GCA GCT CCT CAA ACA GCT CAA |
| ***Nqo1*** | Forward | AGC CCA GAT ATT GTG GCC G |
|  | Reverse | CCT TTC AGA ATG GCT GGC AC |

# Supplementary Figures


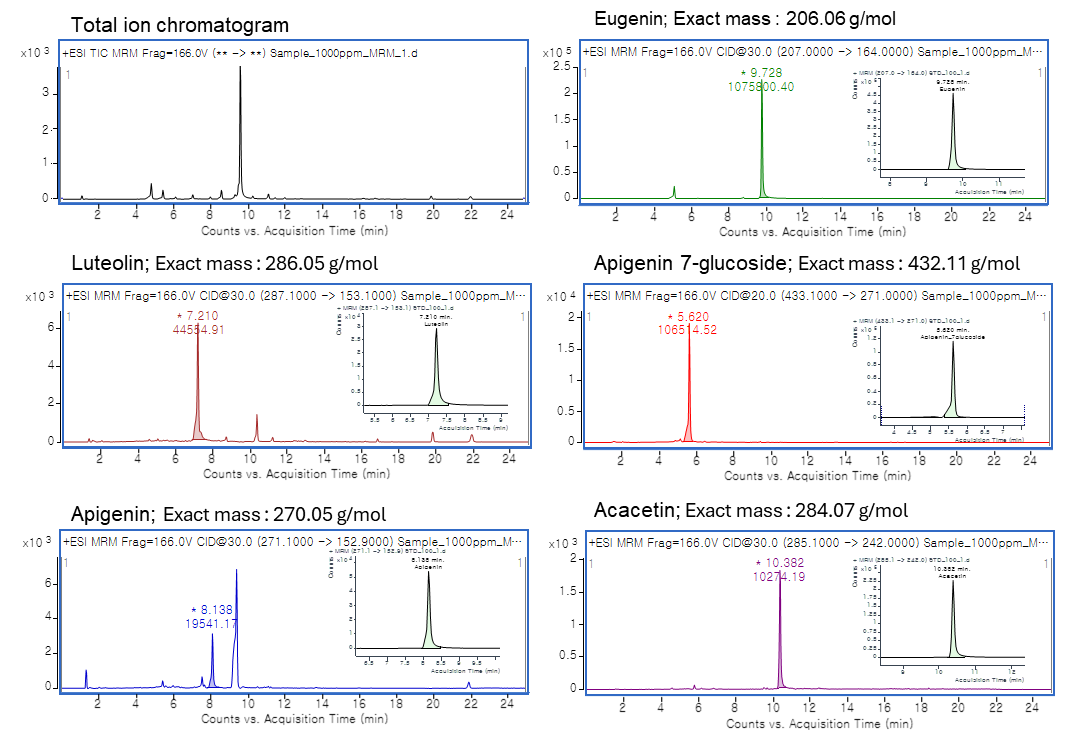


**Supplementary Figure 1. LC-MS/MS analysis of MPSK and bioactive compounds.** The retention time of eugenin was 9.8 min and the content was measured as 44.03 ng/ml. The retention time of luteolin was 7.2 min and the content was measured as 22.61 ng/ml. The retention time of apigenin-7-glucoside was 5.6 min and the content was measured as 16.47 ng/ml. The retention time of apigenin was 8.1 min and the content was measured as 5.97 ng/ml. The retention time of acacetin was 10.4 min and the content was measured as 0.82 ng/ml.


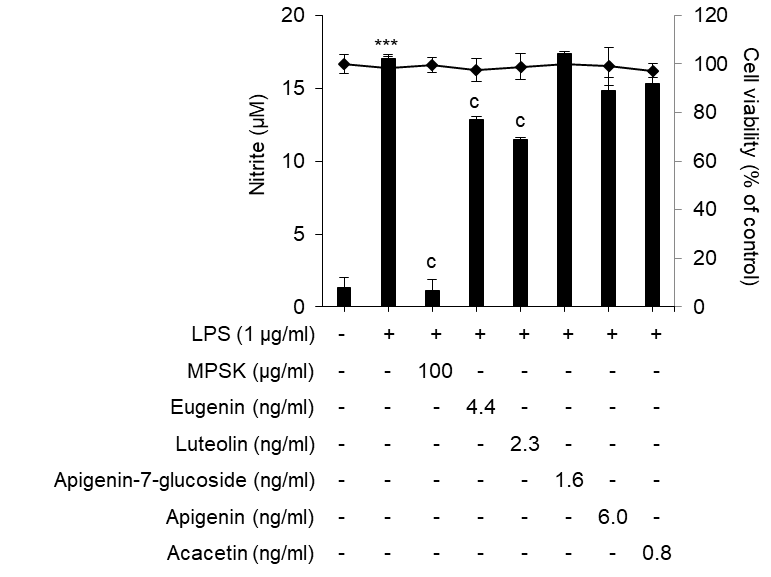


**Supplementary Figure 2. Effects of bioactive compounds of MPSK on NO production and cell viability.** RAW 264.7 macrophages were pretreated with MPSK, eugenin, luteolin, apigenin-7-glucoside, apigenin, and acacetin for 2 h, respectively, and then treated with or without LPS (1 μg/ml) for 22 h. The NO levels secreted in the cell supernatant were evaluated using Griess reagent. Each group was measured relative to the LPS-treated group. The NO levels were calculated according to the standard curve prepared using nitrite standard solution and expressed as a bar graph. The dotted line graph represents the percentage of viable cells compared to the non-treated group. Cell viability was evaluated using EZ-Cytox assay kit. All data represent the mean ± standard deviation (SD) of three independent experiments. ^***^*p* < 0.001 (vs. untreated control); ^c^*p* < 0.001 (vs. LPS-treated group).


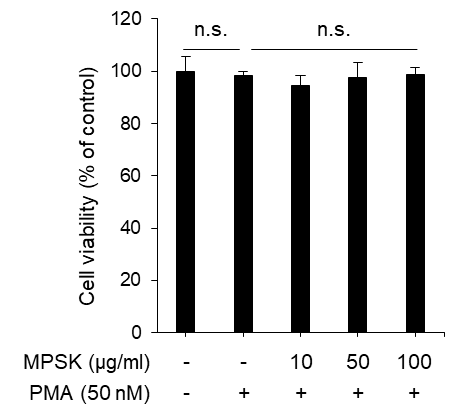


**Supplementary Figure 3. Cytotoxicity of MPSK on HEK 293 cells.** HEK 293 cells were seeded in 96-well plates and pretreated with MPSK for 2 h and then treated with PMA for an additional 22 h. Cell viability was assessed using the EZ-Cytox assay kit. The experiments were performed in triplicate data sets, and the data were expressed as the mean ± SD. n.s., nonsignificant difference.


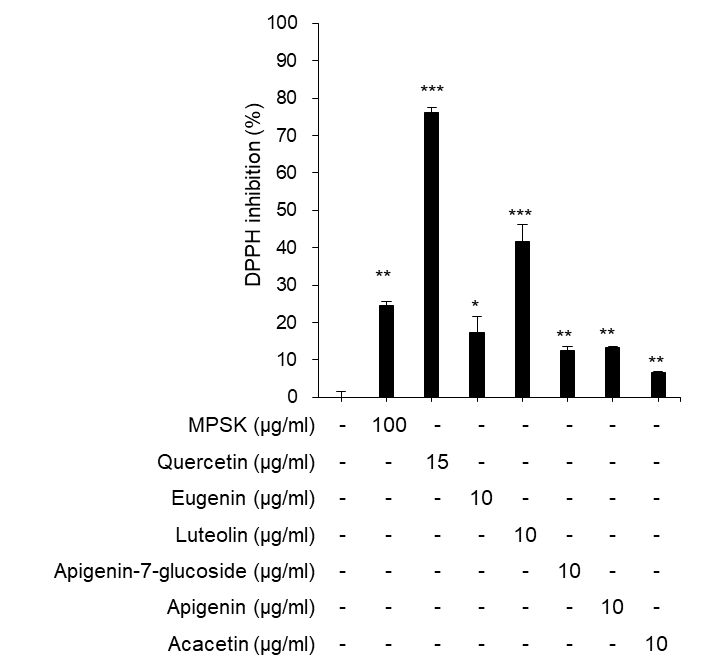


**Supplementary Figure 4. Antioxidant scavenging activity of the bioactive compounds of MPSK through in vitro DPPH assay.** Methanol was used as a negative control and was considered as 0%. Quercetin was used as a positive control. DPPH radical scavenging activity was expressed as the mean ± SD. ^*^*p* < 0.05, ^**^*p* < 0.01, ^***^*p* < 0.001 (vs. untreated control)
